# New insights into the role of macrophages in cancer immunotherapy

**DOI:** 10.3389/fimmu.2024.1381225

**Published:** 2024-03-28

**Authors:** Li Zhou, Tiantian Zhao, Ruzhe Zhang, Chen Chen, Jiwei Li

**Affiliations:** ^1^Department of Pulmonary and Critical Care Medicine, The Second Xiangya Hospital, Central South University, Changsha, Hunan, China; ^2^Research Unit of Respiratory Disease, Central South University, Changsha, Hunan, China; ^3^Diagnosis and Treatment Center of Respiratory Disease, Central South University, Changsha, Hunan, China; ^4^Clinical Medical Research Center for Pulmonary and Critical Care Medicine in Hunan Province, Changsha, China; ^5^Department of Oncology, The Second Xiangya Hospital, Central South University, Changsha, China; ^6^Department of Pathology, Fudan University Shanghai Cancer Center, Shanghai, China

**Keywords:** macrophages, tumor microenvironment, cancer, immunotherapy, PD-L1

## Abstract

Macrophages are the main component of the tumor microenvironment, which are differentiated from monocytes in the blood and play an important role in cancer development. Tumor-associated macrophages (TAMs) can promote tumor growth, invasion, metastasis, and resistance to anti–programmed death receptor 1 therapy by regulating programmed cell death ligand 1 expression and interacting with other immune cells in the tumor microenvironment. However, when activated properly, macrophages can also play an anti-tumor role by enhancing the phagocytosis and cytotoxicity of tumor cells. TAM is associated with poor prognosis and drug resistance in patients treated with immunotherapy, indicating that macrophages are attractive targets for combined therapy in cancer treatment. Combination of targeting TAMs and immunotherapy overcomes the drug resistance and achieved excellent results in some cancers, which may be a promising strategy for cancer treatment in the future. Herein, we review the recent findings on the role of macrophages in tumor development, metastasis, and immunotherapy. We focus mainly on macrophage-centered therapy, including strategies to deplete and reprogram TAMs, which represent the potential targets for improving tumor immunotherapy efficacy.

## 1 Introduction

Cancer is one of the major public health issues worldwide and is the leading cause of death in many countries. According to the latest data published in 2023, approximately 1,958,310 new cancer cases were present in the United States (1). Moreover, due to the high mortality rate and low cure rate of cancer, it has brought heavy economic burden to individuals, families, and society. Therefore, the prevention and treatment of tumors were urgent to further reduce the morbidity and mortality rates. Surgery, radiotherapy, and chemotherapy are three traditional treatment strategies for cancer, but the treatment outcome was still dismal in some patients (1, 2). In recent years, emerging treatment methods have been developed, such as Chimeric antigen receptor (CAR)-T cell therapy and immune-checkpoint inhibitors, which were considered the fourth treatment mode following traditional therapy. At present, immunotherapy has been approved for clinical use, mainly including programmed death receptor 1 (PD-1) inhibitors and CAR-T cell therapy, both of which have achieved excellent results in some advanced stage malignant tumors (3–6). However, the efficacy of PD-1 inhibitor was limited in some patients with cancer (7), and the efficacy needs to be further improved.

The tumor microenvironment was considered to be a key factor affecting tumor progression, metastasis, and treatment results (8, 9). Exploring the tumor microenvironment is the cornerstone of improving the response rate and developing new cancer immunotherapy strategies. In addition, macrophages were reported to be one of the most important immune cells in the tumor microenvironment (9). Based on the function of phagocytosis, macrophages can eliminate tumor cells at an early stage, but, under the stimulation of the stimulating factors in the tumor microenvironment, they gradually transform into tumor-related macrophages with the M2 phenotype and promote tumor growth and metastasis by inhibiting immunity, inducing angiogenesis and supporting cancer stem cells (10). To sum up, it is of great significance to explore in great depth the role of macrophages in the tumor microenvironment, and targeting macrophages may be a promising anti-tumor strategy in the future.

## 2 Origin, polarization, and function of macrophages

Macrophages originate from the monocytes in the circulation, and substantial heterogeneity was observed among each macrophage population (11). According to phenotype and function, macrophages can be divided into two types: classically activated macrophages (M1 macrophages) and alternatively activated macrophages (M2 macrophages) (12). M0 macrophages could differentiate into M1 macrophages under the stimulation of lipopolysaccharide and interferon-γ (IFN-γ), whereas they differentiate into M2 macrophages with the stimulation of interleukin (IL)-4, IL-10, and IL-13 (13). M1 macrophages could produce multiple cytotoxic substances, such as nitric oxide and reactive oxygen species, and thereby activate the function of multiple immune cells and reduce microbial activity, ultimately eliminating microbial infection (14). Meanwhile, a variety of cytokines were produced by M1 macrophages, including tumor necrosis factor–α (TNF-α), growth inhibitors, and anti-angiogenic factors, which could inhibit cancer progression (14). On the contrary, M2 macrophages often function as anti-inflammatory factors by reducing the inflammation response, promoting tissue repair and remodeling the immune system (10, 14). Tumor-associated macrophages (TAMs) were mainly thought to be M2 type in the tumor microenvironment, which could promote tumor growth, invasion, and metastasis.

## 3 Macrophages in the TME promote tumor progression

Macrophages are involved in different stages of tumor development. In the early stage, tumor cells release cytokines and exosomes and attract macrophages and other immune cells into the tumor stroma, where macrophages promote tumor growth, migration, and metastasis (10). As a key component of the tumor microenvironment, macrophages can produce an anti-tumor effect and cause tumor necrosis with powerful swallowing phagocytosis (15), but some studies have shown that TAM is an important driving factor of tumor progression. In the tumors formed, TAM promotes the growth and proliferation of cancer cells, angiogenesis, and lymphangiogenesis and inhibits the immune response of effector T cells (16).

TAM is considered a proinflammatory and anti-tumor phenotype (M1 type) in the early stage of lung cancer and gradually displays an anti-inflammatory and tumor-promoting phenotype in the process of cancer progression (10). TAM could promote tumor development through immune regulation and non-immune processes (17–19). For example, TAM secretes a large number of pro-angiogenic factors such as vascular endothelial growth factor (VEGF) to promote tumor angiogenesis and metastasis (20).

In the tumor microenvironment, macrophages account for half of the total number of tumor cells and are mainly M2 phenotypes. The quantity of macrophages in the tumor microenvironment is associated with tumor micro-vessels and is negatively correlated with the survival outcome in patients with non-small cell lung cancer (NSCLC) (21, 22). In recent years, a growing body of research has revealed the TAM multifaceted regulation of the co-evolving cancer ecosystem based on next-generation technologies and single-cell sequencing technology (12, 22). Therefore, this section mainly introduces the function and mechanism of TAM in tumors.

### 3.1 Anti-tumor effect of M1 type TAM

Inhibition of anti-tumor immunity was reported to be the main pathogenic mechanism of TAM. TAM could downregulate the release of the immunostimulatory factor IL-12, which can trigger the tumor-killing effect of natural killer (NK) cells and cytotoxic CD4+ T cells (23). In addition, many immunosuppressive factors produced by TAM could also mediate cancer development, such as IL-10, transforming growth factor–β, and prostaglandin E2 (10, 24, 25).

TAM can also directly inhibit the function of T cells through specific enzyme activities, such as arginase 1 (ARG1), which is a hydrolase that controls the catabolism of L-arginine. ARG1 is induced by multiple signaling pathways mediated by IL-4, IL-10, and hypoxia and affects T-cell function by limiting the activity of the semi-essential amino acid L-arginine (25). TAM can also promote T-cell apoptosis by inhibiting the expression of programmed cell death ligand 1 (PD-L1) and B7 homolog 1 on T cells (12, 25).

### 3.2 The function of M2 type TAM in promoting tumor development

The function of M2 macrophages in promoting tumor development depends on the proinflammatory cytokines, such as TNF-α, IL-6, and IL-11, which can activate the nuclear factor–κB (NF-κB) and signal transduction and activator of transcription 3 (STAT3) pathway in cancer cells (10, 12, 13, 18, 25). In addition, M2 TAM promoted tumor progression by promoting angiogenesis and lymphangiogenesis by increasing the expression of VEGF-A and VEGF-C (18, 20, 25).

## 4 Macrophages and anti–PD-1/PD-L1 immunotherapy

### 4.1 Effect of TAMs on PD-1/PD-L1 expression

The PD-1/PD-L1 pathway was abnormally activated in various cancers (6, 26), and anti–PD-1/PD-L1 immunotherapy has been widely used or tried in clinical trials in many solid tumors, such as lung cancer, advanced metastatic melanoma, esophagus cancer, and colorectal cancer (27, 28). However, the efficacy of PD-1 inhibitors was still dismal in some patients with high expression of PD-L1, and the concrete mechanisms remain largely unknown.

**Figure 1:**
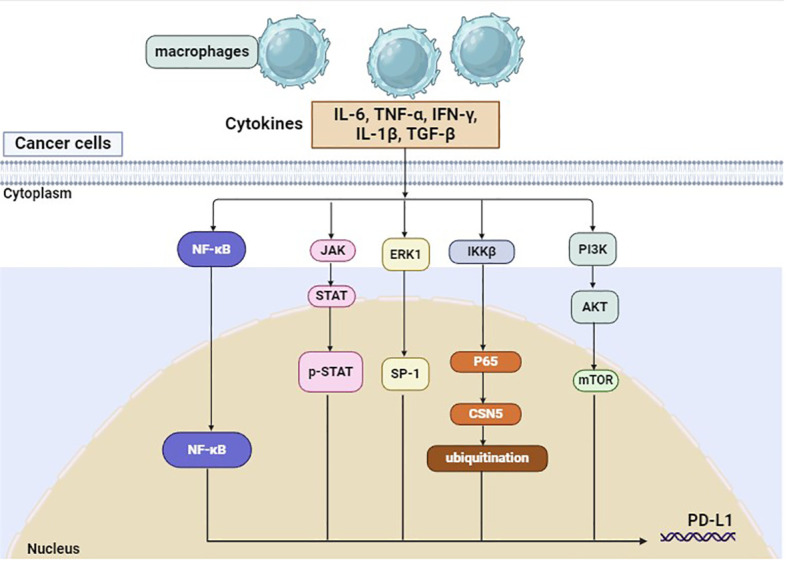
PD-L1 on tumor cells can be regulated by macrophages.

Previous studies have demonstrated that TAMs can regulate the expression of PD-1/PD-L1 through the activation of different signaling pathways (**Figure 1**), which, in turn, affects the efficacy of PD-1/PD-L1 inhibitors. CD163+ TAMs in the tumor microenvironment are reported to be positively correlated to PD-L1 expression in various cancers, including pancreatic cancer and liver cancer. Multiple cytokines released by TAM, including IL-6 and TNF-α, can upregulate PD-L1 expression by activating Janus kinase (JAK)/STAT3, phosphoinositide 3-kinase (PI3K)/AKT, NF-κB, or Extracellular signal-regulated kinase (ERK) 1 and 2 signaling pathways (29, 30). In addition, PD-L1 protein expression could also be upregulated by TNF-α through post-translational regulation (29).

### 4.2 TAMs and anti–PD-1 resistance

In addition to the PD-L1 expression on tumor cells, the tumor microenvironment was also a key factor associated with anti–PD-1 resistance. As mentioned above, cytokines released by TAMs could regulate PD-L1 protein expression, which was reported to be an important predictor for anti–PD≥1/PD≥L1 therapy. In recent years, multiple immune cells have been identified in TME, and the cancer ecosystem has evolved over time, which plays a complex role in cancer development (31, 32). The interaction between macrophages and other immune cells was explored and demonstrated to be correlated to the response to immunotherapy (31). Single-cell and spatial analysis showed that interaction between FAP^+^ fibroblasts and SPP1^+^ macrophages could promote the formation of immune-excluded desmoplasic structures and restrict T-cell which reduces the efficacy of immunotherapy (31). In triple-negative breast cancer, high levels of CXCL13^+^ T cells are associated with the proinflammatory features of macrophages and can predict the clinical benefit of checkpoint inhibitors (32).

Exosomes are small extracellular vesicles that play a crucial role in various cell activities in cancer. Recent studies have reported that macrophage-derived exosomes may promote the formation of a pre-metastatic niche that facilitates tumor growth and metastasis. M2 macrophage–derived EVs can drive anti–PD≥1/PD≥L1 therapy resistance, promote the expression of drug-resistant genes in tumor cells, or affect the immune cell spectrum in TME (33, 34). Therefore, the interaction between TAMs and TME may contribute to anti–PD≥1 therapy resistance in cancer, providing a theoretical basis for the combination use of targeting macrophages and anti–PD≥1/PD≥L1 therapy.

### 4.3 Effect of anti–PD-1/PD-L1 therapy on macrophages

Previous studies have shown that PD-1 inhibitors have an impact on TME in various cancers (35). In non–small cell lung cancer, single-cell RNA sequencing demonstrated that the tumor microenvironment was remodeled after neoadjuvant PD-1 blockade combined with chemotherapy, and TAMs were transformed into a neutral type instead of an anti-tumor phenotype (36). Furthermore, anti–PD-L1 therapy can inhibit tumor growth by reducing PD-L1 expression and promoting the expression of the co-stimulatory molecules CD86 and major histocompatibility complex class II (MHC-II) (37). In addition, the phagocytic ability and immune function of macrophages were also enhanced by anti–PD-L1 therapy, which activates T cells in the TME and eradicates cancer cells (37). Therefore, anti–PD-L1 therapy may repolarize macrophages, enhance the phagocytic ability of macrophages, and ameliorate the tumor microenvironment in some patients.

## 5 Targeting macrophages in the tumor microenvironment

As TAM is involved in tumor immunity and tumor development, it may become a promising target in the future. Current treatment strategies targeting macrophages can be roughly divided into two categories: TAM depletion and TAM reprogramming (**Supplementary Figure 1**). In order to ensure treatment efficacy, targeting TAMs was frequently combined with other treatments in clinical studies, such as immunotherapy, chemotherapy, and radiotherapy (**Table 1**) (38–48).

**Table 1:** Selected clinical trials of agents targeting tumor-associated macrophages.

Compound	Clinical phase	Tumor type	Combination therapy	NCT identifier
Chemokine inhibitors				
Carlumab (anti-CCL2 antibodies; Centocor)	Phase II (completed)	Prostate cancer	NA	NCT00992186
BMS-813160 (CCR2/CCR5 antagonist; Bristol Myers Squibb)	Phase II (completed) [38]	Renal carcinoma	Nivolumab plus ipilimumab	NCT02996110
	Phase I/II (completed)	Pancreatic cancer, CRC, NSCLC	Nivolumab, Nab- paclitaxel	NCT03184870
	Phase II (ongoing)	Hepatocellular carcinoma	Nivolumab	NCT04123379
PF-4136309 (CCR2 antagonist; Pfizer)	Phase II (completed) [39]	PDAC	Nab-paclitaxel, gemcitabine	NCT01413022
CCR5 antagonist (Pfizer)	Phase I (completed) [40]	CRC	Pembrolizumab	NCT03274804
	Phase I (completed)	Pancreatic cancer, CRC	Nivolumab plus ipilimumab	NCT04721301
CSF1R inhibitors				
PLX3397 (Plexxikon)	Phase I/II (ongoing)	Sarcoma, nerve- sheath tumours	Sirolimus	NCT02584647
	Phase I/II (Terminated)	Advanced melanoma and solid tumours	Pembrolizumab	NCT02452424
	Phase I/II (Completed)	Breast cancer	Eribulin	NCT01596751
	Phase I/II (completed) [41]	Glioblastoma	Radiotherapy, temozolomide	NCT01790503
BLZ945 (Novartis)	Phase I/II (Terminated)	Solid tumours	PDR001 (anti- PD1)	NCT02829723
Anti-CSF1R antibodies				
LY3022855 (IMC-C S4; Eli Lilly)	Phase I/II (ongoing)	Melanoma	MEK/BRAF inhibitors	NCT03101254
Emactuzumab (RO5509554/RG7155; Roche)	Phase II (Terminated)	Gynecological neoplasms and ovarian cancer	Gynecological neoplasms and ovarian cancer	NCT02923739
	Phase I/II (ongoing)	PDAC	Nab- paclitaxel, gemcitabine	NCT03193190
	Phase I (completed) [42]	Solid tumors	Paclitaxel	NCT01494688
	Phase I (completed) [43]	Solid tumors	Atezolizumab	NCT02323191
	Phase I (completed) [44]	Solid tumors	RO7009789 (agonist anti-C D40)	NCT02760797
AMG820 (Amgen)	Phase I/II (completed) [45]	Pancreatic cancer, CRC, NSCLC	Pembrolizumab	NCT02713529
ARRAY-382 (Pfizer)	Phase I/II (completed) [46]	Solid tumors	Solid tumors	NCT02880371(1)
Agonist anti-CD40 antibodies				
CP-870,893 (Pfizer; UPenn)	Phase I (completed)	Melanoma	NA	NCT02225002
	Phase I (completed) [47]	Solid tumors	Paclitaxel, carboplatin	NCT00607048
SEA-CD40 (Seagen)	Phase I (ongoing)	Solid and hematological tumors	Pembrolizumab, gemcitabine, Nab-paclitaxel	NCT02376699
Agonist anti-CD40 antibodies (cont.)				
APX005M (Apexigen)	Phase I (ongoing)	Melanoma, renal carcinoma	Nivolumab, ipilimumab	NCT04495257
	Phase I (ongoing)	Melanoma	Pembrolizumab	NCT02706353
	Phase II (ongoing) [48]	Oesophageal cancer	Radiation, paclitaxel, carboplatin	NCT03214250
	Phase I/II (ongoing)	Pancreatic cancer	Nab- paclitaxel, gemcitabine, nivolumab	NCT03214250
RO7009789 (Roche)	Phase I (completed)	Solid tumors	Vanucizumab (anti-A ng2– VEGF bispecific antibody)	NCT02665416
	Phase I (completed)	pancreatic cancer	Nab- paclitaxel and gemcitabine	NCT02588443
CDX-1140 (Roswell Park Cancer Institute)	Phase I (ongoing)	Breast cancer	Radiation, biological therapy, poly-I CLC	NCT04616248
NG-350A adenoviral vector (PsiOxus Therapeutics Ltd)	Phase I (ongoing)	Solid tumors	Immune-checkpoint blockade immunotherapy	NCT05165433

### 5.1 Depletion of TAM

Depletion of macrophages in the tumor microenvironment may be an effective treatment strategy for cancer, either alone or in combination with chemotherapy. Inhibition of the signal transduction axis of colony-stimulating factor-1/colony-stimulating factor-1 receptor (CSF1/CSF1R), which is necessary for macrophage survival, can induce apoptosis of macrophages. On the one hand, inhibition of CSF-1R combined with radiotherapy or chemotherapy can improve T-cell responses. Blockade of CSF1R signaling can effectively deplete the immunosuppressive TAM and then stimulate the CD8+ T-cell response, resulting in prolonged survival in glioblastoma brain tumors (49). At present, CSF1R inhibitors in combination with chemotherapy are being tested in clinical trials in some cancers, such as localized prostate cancer and orthotopic glioblastoma (49, 50). In addition, blocking CSF1/CSF1R can improve the efficacy of a variety of immunotherapies, including CD-40 agonists (51) and PD-1 inhibitors (52).

As TAM was transformed from monocytes, blocking the recruitment of monocytes in the circulation to the tumor site was another method to reduce TAM in the tumor microenvironment. Recruitment of monocytes from bone marrow to the tumor site is dependent on C-C motif ligand 2 (CCL2)-CC chemokine receptor 2 (CCR2) signal transduction (53). Inhibition of CCR2 causes monocyte retention in bone marrow and leads to depletion of monocytes in the peripheral circulation, reduction of monocyte recruitment to the primary tumor sites and metastatic foci, and consequent reduction of TAM number, resulting in tumor shrinkage and survival improvement (54–56).

Other pathways involved in macrophage recruitment include CXCL12-CXCR4 and the angiopoietin 2 (ANG2)–TIE2 axis (57–59). Therefore, depletion of TEM may cause vascular destruction, neutralization of ANG2 may improve the response to vascular VEGFA blockade, and inhibition of TEM recruitment may inhibit tumor growth (60).

### 5.2 Reprogramming of TAM

As macrophages were the main phagocyte and antigen-presenting cell in the tumor, the immune stimulation function of macrophages was lost after the removal of TAMs. Therefore, reprogramming or repolarization of TAM to enhance its anti-tumor function and limit tumor-promoting properties is a more attractive strategy for cancer treatment. For example, in the mouse model of breast cancer, TAM represents the main source of IL-10 and inhibition of IL-10 signal transduction can significantly improve the efficacy of chemotherapy. The IL-10 secreted by TAM inhibits the IL-12 produced by APCs, thereby inhibiting the anti-tumor response of CD8+ T cells induced by paclitaxel and carboplatin (23). In addition, the repolarization of TAM makes it specifically express the proinflammatory cytokine IFN-α, which could activate NK cells and T cells in the tumor environment and significantly slow tumor growth in the mouse model (61). The epigenetic reprogramming of macrophages by inhibiting histone deacetylase (HDAC) can also trigger an immune response in T cells (62, 63). In the breast cancer model, selective class IIa HDAC inhibitor induces the anti-tumor macrophage phenotype, promotes the T-cell immune response, and increases the response to chemotherapy and immune checkpoint inhibitors (62). In addition, the activation of the PI3K signaling in macrophages can drive the immunosuppressive activity in TAM, whereas inhibition of the PI3K pathway can reprogram macrophages enhance T-cell responses (64, 65).

### 5.3 Macrophage cell therapy

CAR-T cells are reported to be effective in hematological malignancies, whereas the efficacy of CAR-T therapy remains dismal in solid tumors, as the entry of T cells into tumors is restrained (66, 67). However, CAR-macrophages (CAR-M) overcome this disadvantage as the macrophages in the TME could be replenished by circulating monocytes. CAR expression could enhance the antigen-dependent functions of macrophages, such as the secretion of cytokines, polarization, enhanced phagocytic ability, and anti-cancer activity (68). CAR-M cells mediate phagocytosis, exhibit M1 functions in a relatively stable way, and exert anti-tumor effects in primary and metastatic tumors (69). Currently, several clinical trials are underway or being developed to evaluate the anti-cancer efficacy of CAR-M in different tumors.

### 5.4 Combination of targeting macrophages and anti–PD-1 therapy in cancer

The combination of targeting macrophages and anti–PD-1 therapy in cancer has been investigated *in vitro* and *in vivo (* 37, 70–72). As we have noted above, repolarization of TAM was considered a promising strategy for cancer treatment, and this approach can potentiate anti–PD-1 therapy efficacy in hepatocellular carcinoma (72). Chemotherapy and radiotherapy may reset macrophages toward an M1 phenotype and improving the efficacy of immunotherapy in cancer (71). Vinblastine can drive the polarization of TAMs to the M1 phenotype by activating NF-κB, increasing CD8+ T-cell populations, and improving the survival outcome of malignant tumor immunotherapy (71). Bi-target treatment such as PD-1–IL-2 cytokine variant (IL2v), which employs anti–PD-1 as a target moiety that is fused into an immuno-stimulatory IL2v, can improve the therapeutic efficacy by reprogramming immunosuppressive TAMs (70). In conclusion, targeting macrophages combined with anti–PD–1 therapy may be a promising strategy to overcome drug resistance in patients with cancer.

## 6 Conclusion

Macrophages are involved in various cell activities in cancer, and the interaction between macrophages and cancer cells or other immune cells is associated with tumor development. As an important part of the tumor microenvironment, TAMs may be a promosing target for cancer treatment. Targeting macrophages alone or combined with radiotherapy, chemotherapy, and immune-checkpoint inhibitors may produce excellent anti-tumor activity. In addition, the upstream and downstream pathways that may regulate the function of macrophages may also serve as therapeutic targets. In particular, the use of genetic engineering to reprogram macrophages to transform tumor-promoting TAM into anti-tumor macrophages is of great clinical application. Although the combination of targeting macrophages and anti–PD-1 therapy in cancer has been tried in clinical trials or preclinical experiments, this treatment approach is still in its infancy and needs further investigation. Stumbling blocks in the transformation and application of TAM-targeted therapy include the diversity and plasticity of mononuclear phagocytes in the TME (73). The dissection of the TME at the single-cell level confirmed the diversity of macrophages and their relationship with other immune cells (22, 31), which provides a rationale to selectively deplete tumor-promoting macrophages and eliminate tumors. The application of macrophage-targeted therapy in cancer is still in its infancy, and the efficacy and tolerance need to be confirmed in more experiments and clinical trials in the future.

## Author contributions

LZ: Writing – original draft. TZ: Writing – original draft, Formal analysis, Data curation, Conceptualization. RZ: Resources, Project administration, Writing – original draft. CC: Methodology, Investigation, Writing – original draft. JL: Writing – review & editing, Visualization, Validation, Supervision, Funding acquisition.

## Supplementary material

Supplementary Figure 1 Targeting macrophages in the tumor microenvironment.
