# Erratum: New insights into the role of macrophages in cancer immunotherapy

**DOI:** 10.3389/fimmu.2024.1427337

**Published:** 2024-05-16

**Authors:** Frontiers Production Office

**Affiliations:** Frontiers Media SA, Lausanne, Switzerland

**Keywords:** macrophages, tumor microenvironment, cancer, immunotherapy, PD-L1

Due to a typesetting error, the original article was published containing several grammatical/language errors which have now been amended.

The publisher apologizes for this mistake.

The original version of this article has been updated.

