# Supplementary figures and images for: New insights into the role of macrophages in cancer immunotherapy
Source: Front Immunol. 2024 Mar 28;15:1381225. doi: 10.3389/fimmu.2024.1381225 (PMC11007015; doi:10.3389/fimmu.2024.1381225)

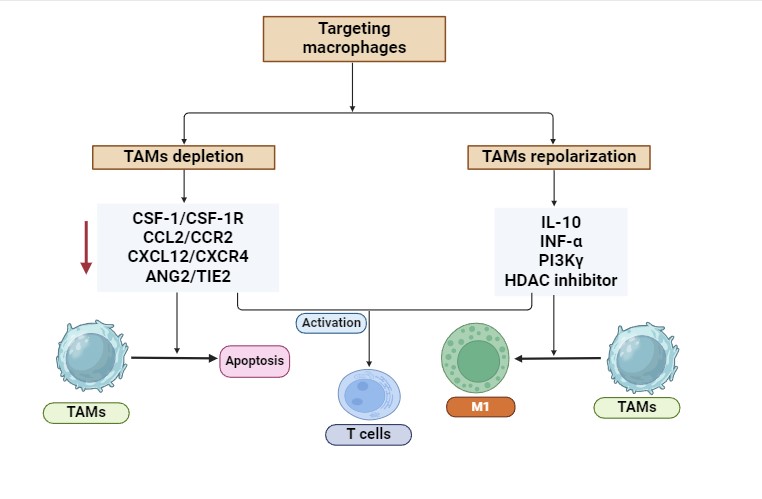

Supplement: Supplementary Figure 1 — Targeting macrophages in the tumor microenvironment. [file Image_1.jpeg]
